# Abstract of Introductory Lecture Delivered at St. Mary's Hospital

**Published:** 1887-10-08

**Authors:** Anderson Critchett

**Affiliations:** Ophthalmic Surgeon, and Lecturer on Ophthalmic Surgery at St. Mary's Hospital


### Abstract of Introductory Lecture delivered at St. Mary's Hospital,

OCTOBER 3RD, 1887,

By Anderson Critchett, M.A. Cantab.,

Ophthalmic Surgeon, and Lecturer on Ophthalmic Surgery at St. Mary's Hospital.

Mr. Anderson Critchett commenced his address with the expression of a sincere hope that the new recruits who were about to join the medical ranks for the first time would regard the professors not as pedagogues, but rather as friends actuated by a single desire to promote their welfare and happiness. Having accurately explained to them the best methods of systematic study, he earnestly exhorted them to diligence. *Labuntur Anni!* In less than three years they would have to put on such an armour of knowledge and expertness as would enable them to face those terrible opponents — Disease and Death. Within the walls of hospitals they might witness the conflict daily, and even there, where the weapons of modern science and treatment were wielded by experienced hands, the foe too often gained the victory. But the time was coming when they must, each of them and alone, commence the struggle. The time was coming when the life of a fellow-creature would, humanly speaking, be given into their hands. When the first-born was in danger, and the mother's eye was turned appealingly to them; when the life-blood was flowing fast from the wounded artery, what would be their feelings if at such a crisis they were found wanting, because the bitter memory of their student-life showed nothing but a barren record of ill-spent hours and wasted opportunities? With calm judgment and unfaltering decision where prompt action was needed, they should seek to combine a gentle, courteous bearing. There should be the iron gauntlet in the velvet glove. Special care was needed in the treatment of children. Their confidence was easily won; nothing was more touching than a child's implicit faith in our efforts for its relief, or more complete than its gratitude for results achieved. Diverging from these topics to others not less worthy of the student's attention, the lecturer contrasted the present with the past, showing what advantages over our predecessors we now enjoy by reason of the greater progress of science. He took a retrospective glance at the history of the medical profession from the middle ages to modern times, paying as he went along a passing tribute of regard to the memories of the various great surgeons and physicians, whose names have shed lustre on the healing art. Coming down to our own day and the triumphs it has witnessed, he alluded to the extraordinary advances made in abdominal surgery, and in the treatment of stone, both in the kidney and bladder, and to the brilliant results of ovariotomy with which the honoured name of Sir Spencer Wells must ever be associated. Then, again, even the opponents of Sir Joseph Lister must allow that the introduction of his system has taught us the incalculable advantages which accrue from extreme care—cleanliness and attention to sanitary laws. Great progress had also been made in the pathology and treatment of diseases of the nervous system; while the comparatively new discoveries in bacteriology opened out an almost illimitable area for research, and might be hopefully relied upon to lead to the downfall of many of those terrible maladies which heretofore baffled their pursuers. Finally, venturing into his own special and favourite province—those Elysian ophthalmic fields, where he had passed his happiest days—Mr. Critchett observed, that he was old enough to remember the introduction of the ophthalmoscope by that great teacher and thinker Helmholtz, who, he rejoiced to say, yet lives to witness the priceless boon which his discovery has conferred upon the human race. It was difficult for those who are now familiar with its use, to conceive the wondering eagerness with which the original workers sought, by the aid of their new weapon, to bring to light those numerous hidden diseases of the eye, which had till then been only partially and most imperfectly recognised. Numerous modifications of the instrument have since been introduced, and among the most recent and most useful improvements has been the ingenious adaptation of the electric light to the ophthalmoscope by his colleague, Mr. Juler. After alluding to the labours of his late father, of the venerated Nestor of English ophthalmic surgery, Sir William Bowman, and of the much lamented Von Graefe, the lecturer reminded his hearers of the colossal work which had been achieved by Professor Donders, who had opened out a new world for thought and investigation, and had elevated the study of practical optics to the dignity of a science. For the rest, gentlemen, said Mr. Crichett in conclusion, I would entreat you to be ever mindful of the dignity of your calling. Never forget the saying of a famous surgeon: "Ours is the noblest of professions but the vilest of trades." It is given to few members of our profession to achieve undying fame. The artist leaves the record of his genius on the painted canvas, and wondering ages worship at his shrine; the poet lives again in words of song that hang upon the lips of countless generations; and the musician breathes his soul into the enchanting melodies that echo for all time. Such fame is not for you. Yours is the priceless luxury of doing good; and in the gratitude of those whose pains you mitigate, whose sorrows you assuage, there lies a richer and a nobler recompense than "proudest record 'mid the tombs of kings." Be therefore of good heart. Rest assured that the reward which waits on diligence will in the fulness of time fall to your lot. As Hamlet says, "If it be not now yet it will come, the readiness is all."

